# Cardiovascular and cardiorespiratory effects of high-intensity interval training in body fat responders and non-responders

**DOI:** 10.1038/s41598-024-65444-z

**Published:** 2024-06-25

**Authors:** Jarosław Domaradzki, Dawid Koźlenia

**Affiliations:** Unit of Biostructure, Faculty of Physical Education and Sport, Wroclaw University of Health and Sport Sciences, al. I.J. Paderewskiego 35, 51-612 Wroclaw, Poland

**Keywords:** High-intensity interval training, School-based intervention, Inter-individual variability, Responders, Non-responders, Body fat, Body mass composition, Physiology, Medical research

## Abstract

This study aimed to investigate cardiovascular and cardiorespiratory adaptations to exercise intervention among participants who showed higher (responders–Rs_BFP_) and lower (non-responders–NRs_BFP_) levels of body fat percentage (BFP) responsiveness. Adolescents (42.5% males) participated in a ten-week school-based high-intensity interval training (HIIT), followed by a comparison of BFP, blood pressure (BP), and cardiorespiratory fitness (CRF). Rs_BFP_ age of 16.15 ± 0.36 years, body height 170.82 ± 8.16 cm, weight 61.23 ± 12.80 kg, and BMI 20.86 ± 3.29 kg/m^2^. Meanwhile, NRs_BFP_ age of 16.04 ± 0.36 years, body height 168.17 ± 8.64 cm, weight 57.94 ± 8.62 kg, and BMI 20.47 ± 2.24 kg/m^2^. HIIT intervention impacted BFP, with a higher decrease in the Rs_BFP_ than the NRs_BFP_ (ΔBFP_Rs_ = − 2.30 ± 3.51(10.34%) vs. ΔBFP_NRs_ = 1.51 ± 1.54(6.96%) *p* < 0.001). The primary comparison showed a statistically significant interaction effect in relation to CRF (F_(1,71)_ = 14.12; *p* < 0.001). Detailed comparisons showed large and significant CRF changes in Rs_BFP_ (7.52%; d = 0.86; *p* < 0.001) but not in NRs_BFP_ (2.01%; d = 0.11; *p* = 0.576). In addition, Rs_BFP_ and NRs_BFP_ benefited equally in SBP (5.49%, d = 0.75; *p* < 0.001; 4.95%, d = 0.74; *p* < 0.001, respectively). These findings highlight that exercise benefits on body fat may be mainly related to gains in CRF. Due to substantial intra-individual variability in adaptation, there is a need for personalized intervention tailored for those with different reaction thresholds in body mass components.

## Introduction

The beneficial effects of physical activity (PA), as a general behavior, on physical, physiological, and mental health have been well-documented^1–6^. Special considerations apply to studying the associations between PA and body mass composition (BMC), resting blood pressure (BP), and cardiorespiratory fitness (CRF). It is widely accepted that PA can drive dependencies between these areas in various ways, but little is known about the role of the individual components. Therefore, multiple analyses, from simple relationships to more complex mediation or pathway analyses, are conducted^7–9^.

The global health problem related to cardiovascular disease (CVD) appears increasingly in childhood and adolescence^5,10^, with increased fat levels a risk factor for elevated BP in adolescents^11^. In contrast, an inverse relationship exists between CRF and BP^12,13^. The triad of associations between these variables involves the relationship between higher CRF and lower or excessive body fat (BF)^14^.

Researchers continue to seek effective ways to fight and prevent diseases associated with excessive adipose tissue and elevated BP. A possible solution may be the introduction of short, very intensive interval exercises in typical physical education (PE) lessons. The advantages and positive effects of this type of intervention are confirmed by documented research results worldwide^15–17^, in Europe^18–22^, and, in recent years, Poland^23–25^. The most common are school-based high-intensity interval training (HIIT) interventions^26–29^. Generally, HIIT is one of the most popular training modalities, recognized as demanding and bringing a lot of positive health adaptations^30^. The evidence from the studies mentioned above generally confirms that HIIT implemented into PE lessons is an effective prophylactic strategy for treating and preventing excessive body weight (particularly BF mass) and elevated BP and improving muscle strength, cardiovascular function, and functional capacity.

The background on the effectiveness of the intervention programs on morphological and physiological features and the exploration of individual responses to exercise stimuli is still an area of growing interest. However, evidence demonstrating the benefits of intensive exercises among children and adolescents has focused on the primary effects. Moreover, a large number of studies focused on average changes caused by exercise intervention (mainly through mean differences between preintervention and postintervention measurements) with little attention (proportionally among the vast majority of studies related to group differences) given to individual variability in measured outcomes^31^. The primary purpose of these studies was to evaluate the extent of variability in response to the interventions. Disparities in responses included benefit, harm, or a neutral effect on an individual, resulting in phenomena described as a poor response, nonresponse, or negative response to the exercise intervention^32^. The most common definition is a lack of outcome change in the expected direction^33^. Some individuals may achieve different effects under the same stimulus, which is related to inter-individual variability in response to exercise training (IVRET)^15,16^. As such, some persons achieve positive benefits, while others have a worsened or unchanged response^18^ and are most commonly called responders (Rs) and non-responders (NRs), respectively. Generally, the origins of variability are separated into technical variability (measurement error), inter-individual variability (between-subject variability), and intra-individual variability (within-subject variability)^31^.

Previous exercise experiments on IVRET mainly presented findings on physiological and biochemical variables related to aerobic and anaerobic capacity (maximum aerobic power and maximum oxygen consumption)^18,33–35^, metabolic markers^16,36–38^ or resting vascular and cardiac functions^19,38,39^. Despite the extensive variation in the exercise protocols studied (e.g., HIIT, moderate-intensity training [MIT], and resistance training) and the use of various groups of participants, such as children and adolescents, young and middle adults, clinical patients, overweight individuals, and prehypertension persons, some threads remain unexplored, and doubts seem unsolved. Indeed, there is a lack of school-based studies conducted under the natural conditions of school PE lessons.

From a public health perspective, implementing intensive physical effort into PE lessons as an antidote to increasing obesity, elevated BP, and decreasing physical efficiency is vital. Furthermore, tailoring individual effort to each participant requires the identification of Rs and NRs in typical school conditions using markers specific for health-related fitness (H-RF) that are easy to assess by teachers.

Most studies have only focused on the prevalence of Rs and NRs^16,38^ or studied individual responses^18,35,39^, and the majority primarily focused on laboratory markers. In addition, little is known about whether participants who adapt to exercise training in one outcome present high or low levels in other outcomes. To the best of the authors’ knowledge, only one article has used such a strategy to date^19^, with the problem evaluated using maximal oxygen consumption (VO_2_ max). Thus, the current study evaluated BF mass, resting BP, and CRF.

This work primarily aimed to investigate cardiovascular and cardiorespiratory adaptations (decreased resting BP and increased CRF) to exercise intervention among participants who showed higher and lower levels of BF responsiveness. Specifically, the aim was two-fold: (1) Do high body fat percentage (BFP) Rs (Rs_BFP_) to HIIT also present significantly greater changes in resting BP and CRF, compared to BFP NRs (NRs_BFP_), that result in higher mean values of the postintervention measurements? (2) Investigate if preintervention BFP explains the inter-individual and intra-individual variability in physiological outcomes. It was hypothesized that participants classified as Rs in terms of BFP would gain more than NRs in cardiovascular and cardiorespiratory measurements, which would be evident in greater mean values after HIIT intervention.

## Material and methods

The current work is part of the project “Physical activity and nutritional education in preventing civilization diseases—theoretical aspects and practical implications for the secondary school physical education program,” which was carried out in a secondary school in Wroclaw (a city in the Lower Silesia region of Poland) over ten weeks. Before running the project, G*Power software (version 3.1.) was used to calculate the a priori sample size. Considering a mixed effect analysis of variance (ANOVA) as the main base analysis, an effect size (ES) of 0.25 (medium ES), a *p*-value of 0.05, a power of 0.80, four groups, and two measurements, the suggested sample size was 179 participants.

Initially, the sample comprised 187 adolescents (66 males aged 16.24 ± 0.34 years and 121 females aged 16.12 ± 0.42 years) from a preselected urban comprehensive secondary school in Wroclaw. Participants were randomly assigned to the experimental (EG) and control (CG) groups. The final study included 141 participants, comprising 52 males (EG n = 31; CG n = 21) aged 16.24 (± 0.34), with a body height of 176.74 cm (± 6.07) and a body mass of 65.42 kg (± 12.51), and 89 females (EG n = 42; CG n = 47) aged 16.12 (± 0.42), with a body height of 164.38 cm (± 6.54), and a body mass of 56.71 kg (± 10.23). Detailed information about participants, procedures, examinations, main statistical characteristics (preintervention and postintervention), detailed percentages of BMI categories, BFP, among others, and statistical comparisons between EG and CG are presented elsewhere^23,25^.

### Participants

The current article is the second study measuring specific anthropometric and physiological features in Rs and NRs to HIIT. Participants comprised 73 EG adolescents, including 31 males and 42 females.

### Procedures

The measurements were recorded before and after the ten-week intervention on the same day between 8:00 a.m. and 1:00 p.m. Participants were asked to excrete, avoid PA and excessive drinking of liquids, and keep their typical morning patterns directly before measurement.

### Measurements

#### Anthropometry and body mass composition

Body height was measured with an accuracy of 0.1 cm using an anthropometer (GPM Anthropological Instruments, DKSH Ltd., Zurich, Switzerland). Body weight and BFP were measured using an InBody230 body composition analyzer (In-Body Co. Ltd., Cerritos, CA, USA). This tool is characterized by very high reliability in males and females, as indicated by high intraclass correlation coefficients (ICCs) for BFP (≥ 0.98), fat mass (FM) (≥ 0.98), and fat-free mass (FFM) (≥ 0.99), and a low standard error of measurement^40^.

#### Resting blood pressure

An Omron BP710, an automatic BP monitor, measured BP. The subjects had to sit quietly for ten minutes before having measurements taken three times at ten-minute intervals. The analyzed results included the means of the three measurements, with SBP and DBP noted.

#### Cardiorespiratory fitness–fitness index

The HST evaluated aerobic capacity, and the results allowed for the calculation of the FI using the following formula^41^: FI = (100 × L)/(5.5 × p), where L = duration of the test in seconds, L < 300 s, and p = heart rate within 1.5 min of the subject stopping the test. The reliability of the HST is acceptable, with an ICC of 0.63^42^.

### Intervention

A PE lesson (total duration of 45 min) started with a standardized ten-minute warm-up of a five-minute slow jog and five minutes of stretching (dynamic and static). The lesson’s main activity was a 14-min Tabata Training Protocol (TAP) comprising three four-minute sessions. Each session’s Tabata protocol consisted of eight cycles of two exercises, including push-ups and high knees. The second session included dynamic lunges and a spider crawl, while the third involved a plank to push-up and side squeeze^23^. Each cycle started with a maximum-intensity exercise lasting 20 s, with participants completing as many repetitions as possible, followed by a ten-second active rest. To verify exercise intensity during the TAP, maximum heart rate (HRmax) was determined with the formula HRmax = 208 − 0.7 × age (16 years). The calculated HRmax (197 bpm) was used to compute the high-intensity exercise ranging from 75 to 80% of HRmax (145–157 bpm). Students’ HRs were monitored during the first TAP PE lesson using a Polar H1 HR monitor (Polar Electro, Kempele, Finland). The monitors were fitted to the student’s chest, level with the xiphoid process, and underneath clothing. HR was displayed on the Polar H1 watch screens during TAP exercises to encourage users to maintain an adequate intensity level. The EG achieved an average HR of 155.8 beats per minute (bpm) (± 18.2; 95% confidence intervals [CIs] 121–184). In subsequent lessons of the Tabata protocol, the exercise intensity of HR measurement was similar to that recorded during the first PE lesson.

### Classification of responders and non-responders

Rs and NRs were defined as individuals who did or did not experience benefits following the completion of the exercise training intervention^43^. Classifying the participants as Rs and NRs based on BFP used the TE, similar to recent studies^16,18^. The following equation was used:

$$TE={SD}_{diff}/\sqrt{2}$$

where TE is the typical error, and SD_diff_ is the standard deviation of the difference (change) between the postintervention and preintervention values.

Rs were classified as participants who demonstrated a greater than two-fold TE from zero decrease in BFP^18^. Thus, the BFP cutoff values were 6.298% for males and 3.245% for females.

### Statistics

The Shapiro–Wilk assessed the normality of data distribution. All quantitative variables were presented as mean, standard deviation, and 95% CIs, while frequencies were presented as numbers and percentages.

Comparisons between baseline variables for sample characteristics of Rs_BFP_ and NRs_BFP_ utilized Student’s unpaired t-test, while analysis of covariance assessed differences in postintervention baseline BFP between Rs_BFP_ and NRs_BFP_, with BFP as a covariate. The flow of BFP in the Rs_BFP_ and NRs_BFP_ between response categories of physiological outcomes was presented with a Sankey diagram. To test differences in frequencies between Rs_BFP_ and NRs_BFP_, a chi-squared test of independence was conducted. The odds ratio (OR) and Cramer’s V for ES were then calculated.

To test changes and disparities in preintervention and postintervention values between groups, a mixed effect ANOVA was conducted, with homoscedasticity and sphericity tested using Levene’s and Mauchly’s tests, respectively (with Greenhouse–Geisser correction when the sphericity assumption was violated). The main effects were *response classification* and *intervention*. Analysis of the Rs_BFP_ and NRs_BFP_ preintervention and postintervention measurements was initially performed by within-subject comparisons and then by between-subject comparisons. Detailed post hoc comparisons used Bonferroni’s correction. A Student’s paired t-test was performed to assess Δ between both response categories. Cohen’s d ES was also calculated.

Linear regression assessed the relationship between preintervention BFP and physiological outcomes when significant changes between respondence categories were observed. Regression equations were calculated using the formula y = b_0_ + b_1_ × BFP (where b_0_ is the constant and b_1_ is the slope). Statistically significant models are presented in figures with regression lines drawn. Then, multiple regression analysis tested the regression constants and slopes. The procedure determines if two regression lines are significantly shifted up or down (test for intercepts) and whether there is an interaction (test for slopes).

To determine if Rs_BFP_ and NRs_BFP_ reacted differently to the HIIT stimulus in physiological outcomes while considering preintervention BFP, the present study assessed intra-individual variability by examining correlation coefficients with repeated measures. To avoid violating the assumptions for linear relationship analysis conducted for repeated measures^44^, the procedure (repeated measure correlation) described by Bakdash and Marusich^45,46^ was used.

The alpha level was fixed at *p* < 0.05 for all tests of statistical significance. Calculations employed Statistica 13.0 (StatSoft Poland 2018, Cracow, Poland) and R software with RStudio (PBC, Boston, MA, USA URL http://www.rstudio.com/ (accessed on 15 May 2023)). Repeated measures analysis was performed with the *rmcorr* package^45,46^.

### Ethical approval

The Senate Research Ethics Committee at the Wroclaw University of Health and Sport Sciences Poland approved this study (consent No. 33/2018 on the 31st October 2018), which followed human experiments’ institutional ethical requirements and the Declaration of Helsinki. The school principal, parents, and study participants gave informed consent before participating. The participants were informed about the study purpose and type, the methods used, and the conditions of their participation. The surveys were conducted by Wroclaw University of Health and Sport Sciences researchers.

## Results

### Baseline sample characteristics

The mean age of Rs_BFP_ was 16.15 years (± 0.36), and the NRs_BFP_ was 16.04 (± 0.36). Rs_BFP_ average height was 170.82 cm (± 8.16), and body weight was 61.23 kg (± 12.80), resulting in a mean body mass index (BMI) of 20.86 (± 3.29). Meanwhile, the NRs_BFP_ mean height was 168.17 cm (± 8.64), their mean body weight was 57.94 kg (± 8.62), and their mean BMI was 20.47 (± 2.24). However, none of the differences between the response categories were statistically significant (*p* > 0.05) (Table 1).

**Table 1:** An overview of participants’ baseline anthropometric and body fat percentage measurements.

Variable	Rs_BFP_				NRs_BFP_				t	*p*	ES
	Mean	-95%CI	95%CI	sd	Mean	-95%CI	95%CI	sd			
Age [years]	16.15	16.04	16.26	0.36	16.18	16.04	16.32	0.36	− 0.34	0.732	0.08
BH [cm]	170.82	168.37	173.27	8.16	168.17	164.82	171.52	8.64	1.32	0.191	0.31
BW [kg]	61.23	57.39	65.08	12.80	57.94	54.60	61.28	8.62	1.20	0.234	0.28
BMI [km/m^2^]	20.86	19.87	21.85	3.29	20.47	19.60	21.33	2.24	0.56	0.579	0.13

Unpaired Student’s t-test results are presented as t-values and *p*-values.

*BH* body height; *BMI* body mass index; *BW* body weight; *CI* confidence interval; *Rs*_*BFP*_ responders; *NRs*_*BFP*_ non-responders; *p p*-value; *sd* standard deviation; *t* t-value.

### Frequencies of responders and non-responders in systolic blood pressure, diastolic blood pressure, and fitness index response categories based on body fat percentage

**Figure 1:**
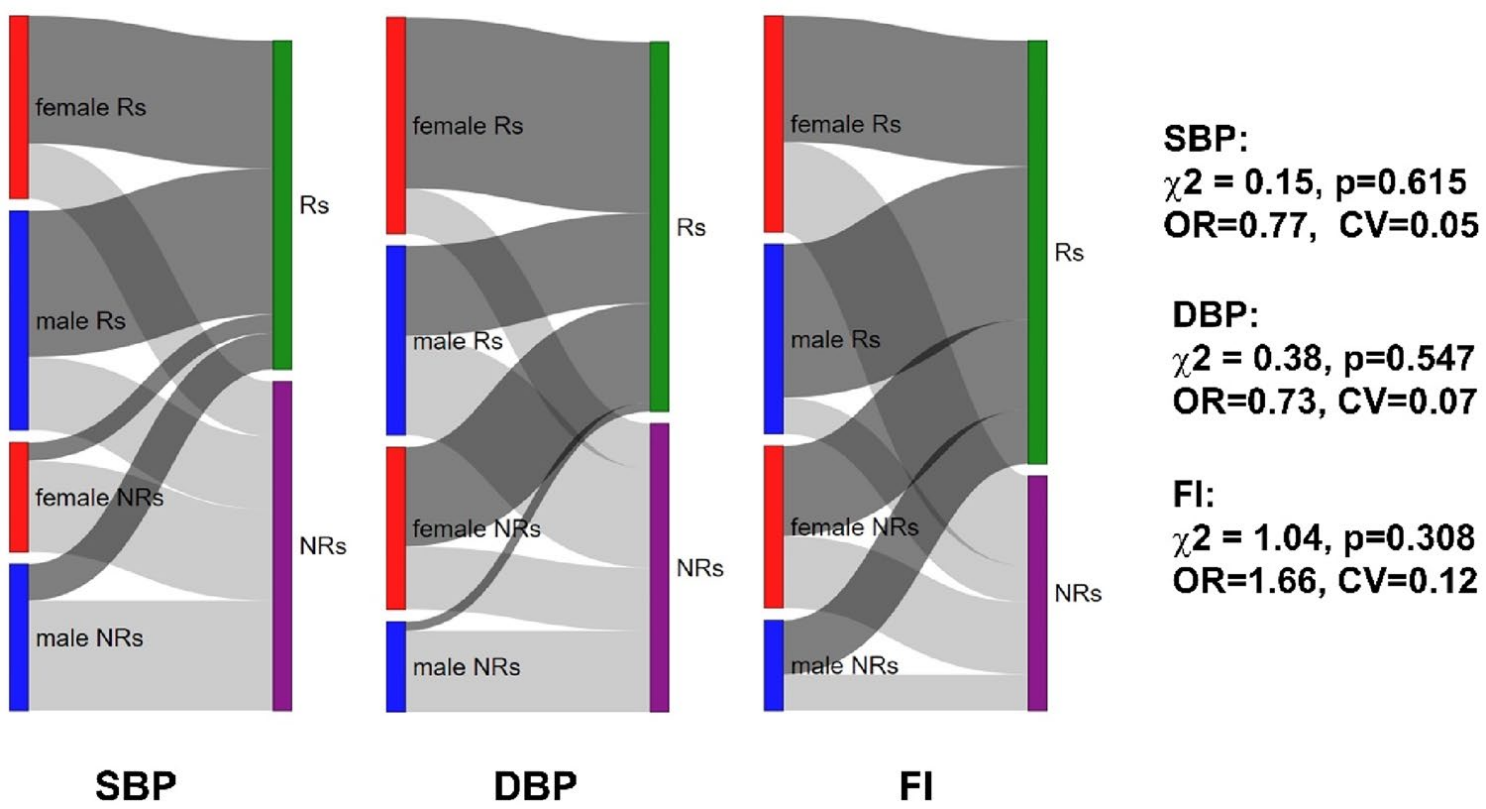
A Sankey diagram showing the switch of body fat percentage responders (Rs) and non-responders (NRs) (males and females) across the systolic blood pressure (SBP), diastolic blood pressure (DBP), and fitness index (FI) response subgroups. Block sizes represent the proportion of sample allocation between subgroups of Rs and NRs. Trajectories highlighted in dark grey belong to the responders to cardiovascular and cardiorespiratory features. Chi-squared independence test statistics are presented on the right.

The potential flow of the Rs_BFP_ in the response categories for all outcomes (systolic BP [SBP], diastolic BP [DBP], and fitness index [FI]) was studied based on BFP. As shown in Fig. 1, most of the Rs_BFPs_ and NRs_BFPs_ (males and females) are located in corresponding SBP, DBP, and FI categories. However, a trend of increasing participation of the NRs_BFP_ in the Rs_BFP_ categories was also observed across subsequent variables (SBP, DBP, and FI). Therefore, any differences in proportions were not statistically significant (*p* > 0.05) (Fig. 1).

### Adaptation in body fat percentage—responder and non-responder characteristics

The baseline BFP in Rs_BFP_ was 22.23% (± 7.97), and NRs_BFP_ was 21.70% (± 8.30), meaning the difference was not statistically significant (*p* > 0.05). The postintervention decrease in the Rs_BFP_ group was − 2.30% (± 3.51), while NRs_BFP_ slightly increased by 1.51% (± 1.54). The difference in changes (Δs) between both respondence categories was statistically significant (*p* < 0.001). Hence, the mean postintervention values between Rs_BFP_ (19.93% [± 8.23]) and NRs_BFP_ (23.21% [± 8.49]) were also significant (*p* < 0.001) (Table 2).

**Table 2:** Baseline, postintervention, and change (Δ) values in body fat percentage responders and non-responders.

Study	Rs_BFP_				NRs_BFP_				*p*	ESb
	Mean	-95%CI	95%CI	sd	Mean	-95%CI	95%CI	sd		
PRE	22.23	19.84	24.62	7.97	21.70	18.49	24.92	8.30	= 0.998	0.06
POST	19.93	17.46	22.40	8.23	23.21	19.92	26.51	8.49	< 0.001	0.39
Δ	− 2.30	− 3.35	− 1.24	3.51	1.51	0.91	2.11	1.54	< 0.001	1.30
Δ%	10.34				6.96					
ESw	0.28				0.18					

The *p*-values are derived from Bonferroni’s post hoc tests (preintervention and postintervention) and Student’s paired t-test (Δs).

*CI* confidence interval; Δ change; Δ% change in %; *Rs*_*BFP*_ responders, *NRs*_*BFP*_ non-responders; *p p*-value; *PRE* preintervention (baseline) measurements; *POST* postintervention measurements; *ESb* effect size between groups; *ESw* effect size within the group.

### High-intensity interval training intervention effects on cardiovascular and cardiorespiratory characteristics

**Figure 2:**
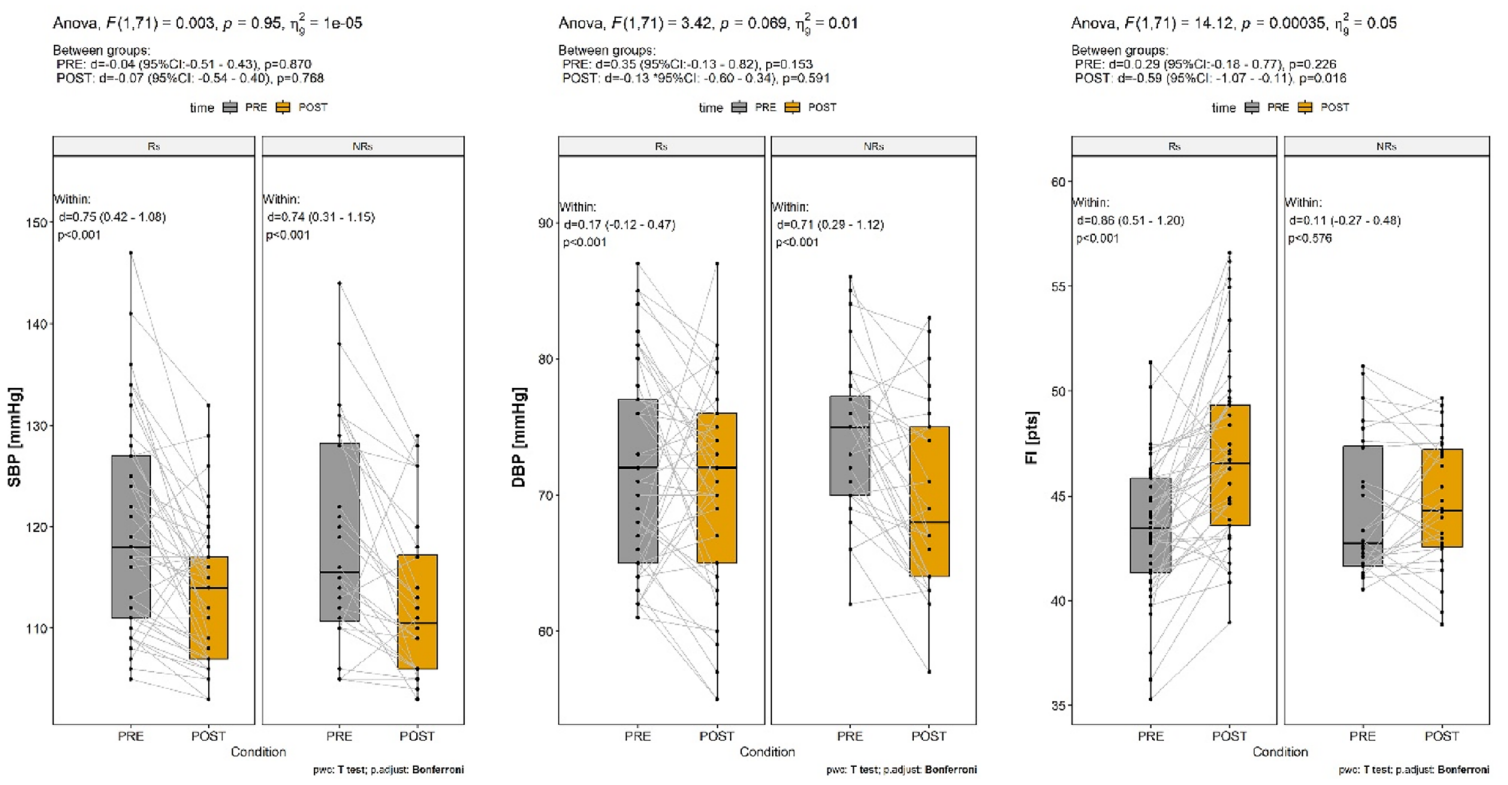
Within and between groups differences between body fat percentage responders and non-responders to the high-intensity interval training intervention in vascular (systolic and diastolic blood pressure) and cardiorespiratory (fitness index) parameters. Mixed effect analysis of variance results in the title of each diagram shows an interaction term (F-value) together with corresponding *p*-values and generalized eta square (ɳ^2^_GES_), which is particularly suitable in a repeated measures design. Besides the effect size derived from the whole model, the within and between-subject comparisons are displayed as Cohen’s d-effect size (d-values with 95% confidence intervals) and *p*-values derived from pairwise Bonferroni post hoc comparisons. The black dots represent the individual values connected to PRE and POST through continuous grey lines. Horizontal solid lines present mean values and boxes are 95% confidence intervals for mean values. *ANOVA* analysis of variance; *CI* confidence interval; *DBP* diastolic blood pressure; *FI* fitness index (cardiorespiratory fitness parameter); *Rs*_*BFP*_ responders, *NRs*_*BFP*_ non-responders; *PRE* preintervention; *POST* postintervention; *SBP* systolic blood pressure.

Table 3 presents characteristics of the preintervention, postintervention, and Δ in measured parameters. Figure 2 provides an overview of the within and between-responder classification adaptations in the vascular and CRF parameters.

**Table 3:** Characteristics of the preintervention, postintervention, and change (Δ) values in systolic blood pressure, diastolic blood pressure, and fitness index between body fat percentage responders and non-responders.

Variable	Study	Rs_BFP_				NRs_BFP_			
		Mean	-95%CI	95%CI	sd	Mean	-95%CI	95%CI	sd
SBP	PRE	119.89	116.45	123.32	11.44	119.04	114.86	123.21	10.76
	POST	113.31	111.22	115.40	6.95	113.14	109.71	116.58	8.86
	Δ	− 6.58	− 9.23	− 3.93	8.83	− 5.89	− 9.09	− 2.69	8.25
	Δ%	5.49				4.95			
DBP	PRE	71.96	69.72	74.19	7.45	73.89	71.01	76.78	7.45
	POST	70.58	68.35	72.81	7.42	70.00	67.00	73.00	7.73
	Δ	− 1.40	− 3.80	1.00	8.00	− 3.89	− 7.39	− 0.40	9.01
	Δ%	1.94				5.27			
FI	PRE	43.64	42.49	44.78	3.82	44.38	42.53	46.23	4.77
	POST	46.91	45.65	48.17	4.19	45.27	43.58	46.96	4.35
	Δ	3.28	1.94	4.61	4.44	0.89	− 0.32	2.10	3.11
	Δ%	7.52				2.01			

Δ change; Δ% change in %; *CI* confidence interval; *DBP* diastolic blood pressure; *FI* fitness index; *Rs*_*BFP*_ responders, *NRs*_*BFP*_ non-responders; *PRE* preintervention (baseline) measurements; *POST* postintervention measurements; *SBP* systolic blood pressure; *sd* standard deviation; *ESb* effect size between groups; *ESw* effect size within the group.

The linear mixed effect model (responder classification × time) revealed a significant interaction effect in increasing FI (F(1,71) = 14.12; *p* < 0.001), and there was also a main effect of time (F(1,71) = 19.26; *p* < 0.001). Considering the Bonferroni adjusted *p*-values, pairwise comparison between time points (pre- vs. post-) showed significant and large FI changes in the Rs_BFP_ (*p* < 0.001; d = 0.82), but not in the NRs_BFP_ (*p* = 0.576; d = 0.13) (Fig. 2). Detailed pairwise comparisons (with Bonferroni’s post hoc correction applied) between groups (Rs_BFP_ vs. NRs_BFP_) showed a significant and moderate disparity in postintervention FI (*p* = 0.016; d = − 0.59) but not in baseline values (*p* = 0.226; d = 0.29). There was no statistically significant interaction between response category and time for both cardiovascular parameters (SBP: F(1,71) = 0.03, *p* = 0.95; DBP: F(1,71) = 3.42, *p* = 0.069). However, a main effect for time was observed in reducing SBP (F(1,71) = 38.68; *p* < 0.001) and DBP (F = 11.40; *p* = 0.001). Detailed post hoc (Bonferroni’s correction applied) within-group comparisons indicated substantial (although small in the case of DBP in Rs_BFP_) benefits in Rs_BFP_ (SBP: *p* < 0.001, d = 0.75; DBP: *p* < 0.001, d = 0.75) and NRs_BFP_ (SBP: *p* < 0.001, d = 0.17; DBP: *p* < 0.001, d = 0.71) (Fig. 2).

### Inter-individual differences in the relationship between preintervention body fat percentage and changes in cardiorespiratory fitness of the responders and non-responders

Inter-individual variability in the relationship between changes in physiological outcomes and preintervention BFP was studied using linear regression. Linear modeling indicated the different potential of BFP at baseline in influencing the cardiovascular parameters and CRF. Baseline BFP did not significantly influence SBP or DBP, as there was no marked shift between regression lines (SBP: b = − 0.04, *p* = 0.961; DBP: b = − 1.67, *p* = 0.069). These findings suggest equal changes in SBP and DBP in relation to preintervention BFP in both response categories. Similar slopes between groups confirmed a non-significant interaction term (SBP: b = − 0.17, *p* = 0.507; DBP: b = − 0.02, *p* = 0.093).

**Figure 3:**
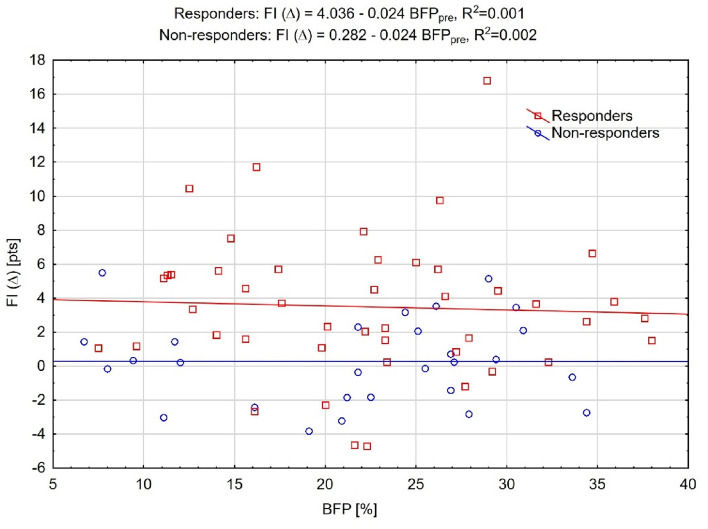
Fitness index (FI) change (Δ) regression based on baseline body fat percentage (BFP). Red squares indicate individual responders’ data, while blue circles indicate non-responders.

There was a significant difference in intercepts of the FI relationship to BFP in both response categories (b = − 1.62; *p* < 0.001) (Fig. 3), though a significant vertical shift showed that the change was higher in Rs_BFP_ compared to NRs_BFP_ based on preintervention BFP. However, non-significant interactions (b = − 0.02; *p* = 0.827) suggested that one-unit changes in the predictor were associated with similar mean response changes in Rs_BFP_ compared to NRs_BFP_.

### Intra-individual variability in fitness index in response to high-intensity interval training based on body fat percentage

**Figure 4:**
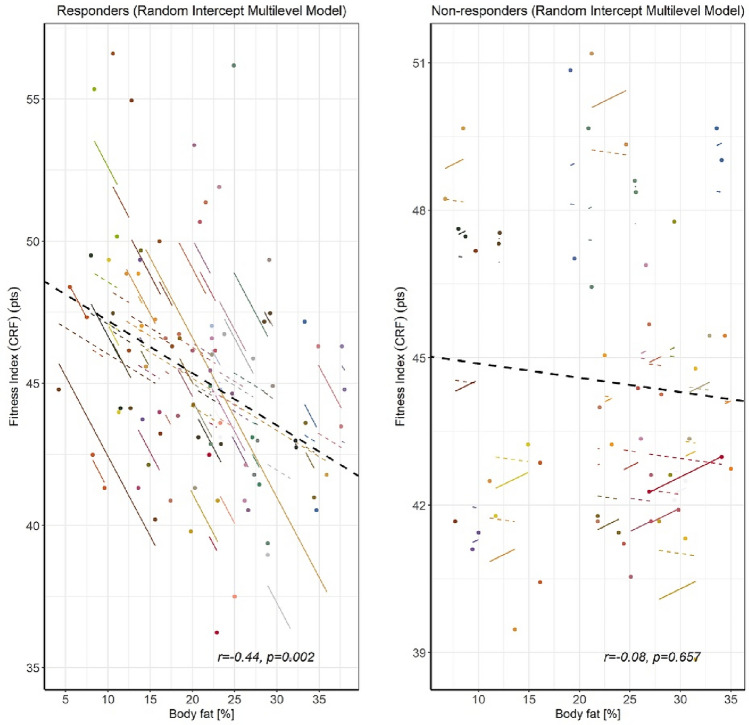
A scatterplot with multiple groups (responders and non-responders at two time points) displaying the relationship between increased body fat percentage (preintervention and postintervention measurements) and changes (decrease or increase) in physiological outcomes. Individual variations are represented by solid regression lines (*rmcorr* fit for each participant), while dashed grey lines represent common general trends in intra-individual variability, which were calculated from a multilevel mixed model with random intercepts and fixed slopes. Graphs are supplemented with r_rm_-values and their corresponding *p*-values.

Repeated measures correlation analysis revealed different associations between the investigated variables in both response categories. The strongest (significant and negative) relationship was found for BFP and FI (r_rm_ = − 0.44; *p* = 0.002) in Rs_BFP_, whereas the same relationship was lowest and non-significant in NRs_BFP_ (r_rm_ = − 0.08; *p* = 0.567) (Fig. 4). Also, decreasing BF was moderately and positively related to decreasing SBP in Rs_BFP_ (r_rm_ = 0.39; *p* = 0.007) and poorly aligned with decreased DBP (r_rm_ = 0.22; *p* = 0.007). In addition, there were moderate negative correlations between BFP and SBP (r_rm_ = − 0.43; *p* = 0.018) and DBP (r_rm_ = − 0.48; *p* = 0.008) in NRs_BFP_.

## Discussion

To the best of the authors’ knowledge, this is the first study to investigate differences in cardiovascular parameters and CRF in Rs_BFP_ and NRs_BFP_ in a school-based HIIT intervention, although the strategy was similar to the approach of Maturana et al.^19,39^. Our results showed that HIIT during one PE lesson per week for ten weeks impacted participants’ BF in various ways, leading to substantial individual variability in Rs_BFP_ and NRs_BFP_. Rs had a greater increase in CRF (large effect), evidenced by higher final postintervention levels (moderate effect), compared to NRs, though there were no differences in the number of changes between both response categories, including for BP parameters. Both categories decreased in SBP after HIIT, while only NRs did for DBP. Inter-individual analysis of the relationship between preintervention BF and changes in physiological outcomes showed similar individual variability in both response categories, resulting in a lack of interaction between categories. In addition, the variability in any outcome was not associated with initial BF mass in either category, indicated by the constant, horizontal regression line, slopes, and coefficients. The only significant effect was an association between BF at baseline and FI responsiveness in Rs_BFP_. Additionally, the BF response increase in pre and post-differences was accompanied by positive changes (increasing differences) in BP parameters and FI. Meanwhile, the opposite relationship was observed for NRs_BFP_ for BP and FI.

Findings support the latest exercise guidelines for individuals with overweight/obesity, emphasizing the benefits of a multicomponent exercise approach for cardiometabolic health. Multicomponent exercise, including bodyweight drills and resistance-based activities, may enhance musculoskeletal fitness in inactive, middle-aged adults with overweight/obesity^47,48^. For children, a multidimensional physical activity intervention could also be effective in improving musculoskeletal and cardiovascular fitness, and the term HIIT seems to be effective^49^.

We observed the impact of HIIT on fat tissue reduction. There was such substantial individual variability that it was possible to distinguish Rs and NRs based on BFP. Study conducted by Dominic and Kishore^50^ showed the effectiveness of HIIT on BF reduction, which was confirmed in a meta-analysis by Khodadadi et al.^51^. With joint improvement in body composition, increased CRF^52^ and motor fitness^53^ are observed. However, multidirectional analyses are less common^54^. The benefits of HIIT are often achieved in a few areas, though there is a lack of deeper insight into individual responses, which may differ due to physiological processes^55^.

Despite the mean results confirming improvement in some parameters, some subjects did not respond or had a varied effect in their response magnitude^56^. Therefore, the question arises of how to produce beneficial gains in NRs, particularly since the observations showed inconsistent results. Juric et al.^57^ found high variability in body tissue despite a significant impact on CRF after HIIT intervention. Nonetheless, the lack of consistency in observations should be explained by inter-individual analyses. A study by Montero and Lundy^43^ suggested that non-reactive subjects should receive a higher intervention load. On the other hand, a high dose of any PA may lead to overfatigue or overtraining^58^. Moreover, adolescents may reject PA if the intensity and/or volume is too high^59^. As such, it is essential to start from an appropriate level and then increase the load over time^60^. Our study showed that improvement in BFP was associated with better CRF, which was also observed in studies by Lan et al.^61^ and Guo et al.^62^. Generally, aerobic metabolic processes use fatty acids, which may explain the links between losing BF and joint improvement and increased CRF^63^.

The methodological approach used to study HIIT effects on BP and CRF in Rs_BFP_ and NRs_BFP_ was unusual, meaning that comparing the results to other works is challenging. The same approach was presented by Maturano^19,39^, though they examined variability in H-RF parameters by measuring VO_2_ max. Their findings demonstrated the influence of exercise intensity and biological variability on an individual’s V̇O_2_ max response after MIT and HIIT, highlighting the importance of personalizing interventions^64^.

Individualization of training intervention by load optimization, despite the target (sports results or health), is an effective way to achieve goals^19,39,64^. Training modifications are needed when progression is not visible, though appropriate increases must be introduced^65^. From a practical point of view, addressing the training intervention on an individual level is challenging when targeted at a population. However, their effectiveness could grow when the factors distinguishing individual responses are identified^66^. Baker et al.^67^ showed that body composition influences individual intervention responses. Also, a study by Andrade-Mayorga et al.^68^ showed that physiological characteristics impacted changes. These observations demonstrate that factors modify individual responses based on physical variables. As such, some of them should be the basis for PA program development.

Another aspect was the analysis of inter-individual variation in the relationship between changes in physiological outcomes and preintervention BF. Intra-individual variability was similar in Rs_BFP_ and NRs_BFP_, with no dependence of baseline BFP on changes in SBP, DBP, and FI. Similar studies conducted by Maturano^39^ (for other variables) found relationships between both response categories, with more substantial increases in peak power output, lactate threshold, and microvascular responsiveness in Rs, whereas a more significant increase in cycling efficiency was observed in NRs. In addition to differences in V̇O_2_ max, a greater increase in microvascular responsiveness was observed in Rs compared to NRs. Furthermore, Rs and NRs did not exhibit differences in metabolic adaptations. As such, there is a growing need for personalized training plans based on the desired clinical outcome.

A study on intra-individual variation in the relationship between differences in BF and physiological outcomes showed varied association patterns in Rs_BFP_ and NRs_BFP_. Rs_BFP_ had positive gains in BF and improvement in all physiological outcomes, while the NRs_BFP_ did not display such an association. There are few methods for studying such a relationship, as the individual response may be due to physiology, hormone levels, and muscle tissue type. As such, creating a multivariable profile of individuals to find the best-fitting training program for achieving optimal physical outcome improvements is required.

The current study was not cross-over in design, which would have provided more robust data. Another limitation was the small number of participants separated into Rs and NRs, which forced sex agglomeration. Indeed, analyzing sexes separately would be more advantageous when inferring the findings. A further limitation is the criteria for identifying Rs and NRs, which were defined by a change in the typical error (TE) of the main outcomes and co-variables from baseline to follow-up (null hypothesis testing). Future analysis should include prior knowledge (distribution) on the intervention’s effects and could apply (combine) the Bayesian method (ROPE + HDI decision-making in posterior distribution) to identify the response categories, an approach that was verified as considerably more advantageous^19,69^. Furthermore, using VO_2_ max to assess CRF would have provided more precise information on adaptation than the Harvard Step Test (HST) method used. Also, using a DEXA device for body morphology measurements would provide more accurate data due to its reference method in body morphology measurements. There is also a need to verify the effectiveness of interventions tailored to non-responders groups. Also interesting would be the identification of factors that affect the response after HIIT.

## Conclusions

Our study showed the effectiveness of school-based HIIT in decreasing BF mass, though there was substantial individual variation. Also, BFP response categories differed from those for other physiological outcomes. Therefore, adaptation in body mass composition does not guarantee a positive response in physiological parameters.

Our findings highlight BF adaptation and benefits from intensive exercise intervention during PE lessons, which may be related to gains in CRF, although BP did not have the same response. However, there were visible positive changes in BP parameters independent of the BF response, though the BF changes may appear before other health-related outcomes are modified. There was substantial intra-individual variability in adaptation. Therefore, there is a need for personalized interventions (structure and load) tailored for persons with different reaction thresholds in body mass components.

## Data Availability

The datasets generated during and/or analyzed throughout the current study are available from the corresponding author upon reasonable request.

## References

[1] Warburton DE, Nicol CW, Bredin SS (2006). Health benefits of physical activity: The evidence. Can. Med. Assoc. J..

[2] Nocon M, Hiemann T, Müller-Riemenschneider F, Thalau F, Roll S, Willich SN (2008). Association of physical activity with all-cause and cardiovascular mortality: A systematic review and meta-analysis. Eur. J. Cardiovasc. Prev. Rehabil..

[3] Nikander R, Sievänen H, Heinonen A, Daly RM, Uusi-Rasi K, Kannus P (2010). Targeted exercise against osteoporosis: A systematic review and meta-analysis for optimising bone strength throughout life. BMC Med..

[4] Lee C, Folsom AR, Blair SN (2003). Physical activity and stroke risk a meta-analysis. Stroke.

[5] Stenman M, Pesola A, Laukkanen A, Haapala E (2017). Effects of two-week high-intensity interval training on cognition in adolescents – a randomized controlled pilot study. Hum. Mov..

[6] Faulkner J, O’Brien WJ, McGrane B, Wadsworth D, Batten J, Askew CD (2021). Physical activity, mental health and well-being of adults during initial COVID-19 containment strategies: A multi-country cross-sectional analysis. J. Sci. Med. Sport.

[7] Stratton G, Canoy D, Boddy LM, Taylor SR, Hackett AF, Buchan IE (2007). Cardiorespiratory fitness and body mass index of 9–11-year-old English children: A serial cross-sectional study from 1998 to 2004. Int. J. Obes..

[8] Pérez-Bey A, Segura-Jiménez V, Fernández-Santos J, Esteban-Cornejo I, Gómez-Martínez S, Veiga OL, Marcos A, Ortega FB, Castro-Piñero J (2019). The influence of cardiorespiratory fitness on clustered cardiovascular disease risk factors and the mediator role of body mass index in youth: The UP&DOWN study. Pediatr. Diabetes.

[9] Domaradzki J, Koźlenia D, Popowczak M (2022). The mediation role of fatness in associations between cardiorespiratory fitness and blood pressure after high-intensity interval training in adolescents. Int. J. Environ. Res. Public Health.

[10] Högström G, Nordström A, Nordström P (2014). High aerobic fitness in late adolescence is associated with a reduced risk ofmyocardial infarction later in life: A nationwide cohort study in men. Eur. Heart J..

[11] GBD 2016 Risk Factors Collaborators. Global, regional, and national comparative risk assessment of 84 behavioural, environmental and occupational, and metabolic risks or clusters of risks, 1990–2016: A systematic analysis for the Global Burden of Disease Study 2016. *Lancet* 390, 1345–1422 (2017). 10.1016/S0140-6736(17)32366-8 PMC5614451 28919119

[12] Agostinis-Sobrinho C, Ruiz JR, Moreira C, Abreu S, Lopes L, Oliveira-Santos J, Mota J, Santos R (2018). Cardiorespiratory fitness and blood pressure: A longitudinal analysis. J. Pediatr..

[13] Kvaavik E, Klepp KI, Tell GS, Meyer HE, Batty GD (2009). Physical fitness and physical activity at age 13 years as predictors of cardiovascular disease risk factors at ages 15, 25, 33, and 40 years: Extended follow-up of the Oslo Youth Study. Pediatrics.

[14] Mintjens S, Menting MD, Daams JG, van Poppel M, Roseboom TJ, Gemke R (2018). Cardiorespiratory fitness in childhood and adolescence affects future cardiovascular risk factors: A systematic review of longitudinal studies. Sports Med..

[15] Bouchard C, Blair SN, Church TS, Earnest CP, Hagberg JM, Häkkinen K (2012). Adverse metabolic response to regular exercise: Is it a rare or common occurrence?. PLoS One.

[16] Alvarez C, Ramírez-Campillo R, Ramírez-Vélez R, Izquierdo M (2017). Effects of 6-weeks high-intensity interval training in schoolchildren with insulin resistance: Influence of biological maturation on metabolic, body composition, cardiovascular and performance non-responses. Front. Physiol..

[17] Delgado-Floody P, Latorre-Román P, Jerez-Mayorga D, Caamaño-Navarrete F, García-Pinillos F (2019). Feasibility of incorporating high-intensity interval training into physical education programs to improve body composition and cardiorespiratory capacity of overweight and obese children: A systematic review. J. Exerc. Sci. Fit..

[18] Bonafiglia JT, Rotundo MP, Whittall JP, Scribbans TD, Graham RB, Gurd BJ (2016). Inter-individual variability in the adaptive responses to endurance and sprint interval training: A randomized cross-over study. PLoS One.

[19] Maturana FM, Soares RN, Murias JM, Schellhorn P, Erz G, Burgstahler C, Widmann M, Munz B, Thiel A, Nieß AM (2021). Responders and non-responders to aerobic exercise training: Beyond the evaluation of VȮ_2max_. Physiol. Rep..

[20] Batrakoulis A, Jamurtas AZ, Metsios GS, Perivoliotis K, Liguori G, Feito K (2022). Comparative efficacy of five exercise types on cardiometabolic health in overweight and obese adults: A systematic review and network meta-analysis of randomized controlled trials. Circ. Cardiovasc. Qual. Outcomes.

[21] Ortega FB, Ruiz JR, Castillo MJ, Sjöström M (2008). Physical fitness in childhood and adolescence: A powerful marker of health. Int. J. Obes..

[22] Rodríguez-Fernández A, Lago Á, Ramirez-Campillo R, Sánchez M, Sánchez-Sánchez J (2023). Cardiopulmonary- versus neuromuscular-based high-intensity interval training during a pre-season in youth female basketball players. Hum. Mov..

[23] Popowczak M, Rokita A, Koźlenia D, Domaradzki J (2022). The high-intensity interval training introduced in physical education lessons decrease systole in high blood pressure adolescents. Sci. Rep..

[24] Domaradzki J, Rokita A, Koźlenia D, Popowczak M (2021). Optimal values of body composition for the lowest risk of failure in Tabata Training’s effects in adolescents: A pilot study. BioMed. Res. Int..

[25] Domaradzki J, Koźlenia D, Popowczak M (2022). Prevalence of positive effects on body fat percentage, cardiovascular parameters, and cardiorespiratory fitness after 10-week high-intensity interval training in adolescents. Biology.

[26] Eddolls W, McNarry MA, Stratton G, Winn C, Mackintosh KA (2017). High-intensity interval training interventions in children and adolescents: A systematic review. Sports Med..

[27] Buchan DS, Ollis S, Young JD, Cooper SM, Shield JP, Baker JS (2013). High intensity interval running enhances measures of physical fitness but not metabolic measures of cardiovascular disease risk in healthy adolescents. BMC Public Health.

[28] Dunstan DW, Daly RM, Owen N, Jolley D, de Courten M, Shaw J (2002). high-intensity resistance training improves glycemic control in older patients with type 2 diabetes. Diabetes Care.

[29] Cano-Montoya J, Ramírez-Campillo R, Martínez C, Sade-Calles F, Salas-Parada A, Álvarez C (2016). Interacción entre farmacoterapia hipotensiva y terapia con ejercicio físico requiere regulación farmacológica en pacientes hipertensos. Rev. Méd. Chile.

[30] A'Naja MN, Reed R, Sansone J, Batrakoulis A, McAvoy C, Parrott MW (2024). 2024 ACSM worldwide fitness trends: Future directions of the health and fitness industry. ACSM's Health Fit. J..

[31] Hecksteden A, Kraushaar J, Scharhag-Rosenberger F, Theisen D, Senn S, Meyer T (2015). Individual response to exercise training - a statistical perspective. J. Appl. Physiol..

[32] Whipple MO, Schorr EN, Talley KMC, Lindquist R, Bronas UG, Treat-Jacobson D (2018). Variability in individual response to aerobic exercise interventions among older adults. J. Aging Phys. Act..

[33] Bouchard C, Rankinen T (2001). Individual differences in response to regular physical activity. Med. Sci. Sports Exerc..

[34] Hopkins WG.How to interpret changes in an athletic performance test. http://www.sportsci.org/jour/04/wghtests.htm

[35] Hecksteden A, Pitsch W, Rosenberger F, Meyer T (2018). Repeated testing for the assessment of individual response to exercise training. J. Appl. Physiol..

[36] Ahtiainen JP, Sallinen J, Häkkinen K, Sillanpää E (2020). Inter-individual variation in response to resistance training in cardiometabolic health indicators. Scand. J. Med. Sci. Sports.

[37] Kozioł K, Zebrowski J, Betlej G, Bator E, Czarny W, Bajorek W, Czarnota B, Czaja R, Król P, Kwiatkowska A (2020). Changes in γH2AX and H4K16ac levels are involved in the biochemical response to a competitive soccer match in adolescent players. Sci. Rep..

[38] Álvarez C, Ramírez-Campillo R, Cristi-Montero C, Ramírez-Vélez R, Izquierdo M (2018). Prevalence of non-responders for blood pressure and cardiometabolic risk factors among prehypertensive women after long-term high-intensity interval training. Front. Physiol..

[39] Maturana FM, Schellhorn P, Erz G (2021). Individual cardiovascular responsiveness to work-matched exercise within the moderate- and severe-intensity domains. Eur. J. Appl. Physiol..

[40] McLester CN, Nickerson BS, Kliszczewicz BM, McLester JR (2020). Reliability and agreement of various InBody body composition analyzers as compared to dual-energy X-ray absorptiometry in healthy men and women. J. Clin. Densitom. Off. J. Int. Soc. Clin. Densitom..

[41] Bajaj, A., Appadoo, S., Bector, C., Chandra, S. Measuring physical fitness and cardiovascular efficiency using harvard step test approach under fuzzy environment. ASAC 29, (2008).

[42] Burnstein BD, Steele RJ, Shrier I (2011). Reliability of fitness tests using methods and time periods common in sport and occupational management. J. Athletic Train..

[43] Montero D, Lundby C (2017). Refuting the myth of nonresponse to exercise training: ‘non-responders’ do respond to higher dose of training: Trainability and exercise dose. J. Physiol..

[44] Bland JM, Altman DG (1995). Statistics notes: calculating correlation coefficients with repeated observations: Part 1–correlation within subjects. BMJ.

[45] Bakdash JZ, Marusich LR (2017). Repeated measures correlation. Front. Psychol..

[46] Bakdash, J. Z. & Marusich, L. R. 2020. rmcorr: Repeated measures correlation. R package version 0.4.1. Available Online at: https://CRAN.R-project.org/package=rmcorr. Accessed 16 April 2021.

[47] Batrakoulis A, Jamurtas AZ, Tsimeas P, Poulios A, Perivoliotis K, Syrou N, Papanikolaou K, Draganidis D, Deli CK, Metsios GS, Angelopoulos TJ, Feito Y, Fatouros IG (2023). Hybrid-type, multicomponent interval training upregulates musculoskeletal fitness of adults with overweight and obesity in a volume-dependent manner: A 1-year dose-response randomised controlled trial. Eur. J. Sport Sci..

[48] Batrakoulis A, Jamurtas AZ, Metsios GS, Perivoliotis K, Liguori G, Feito Y, Riebe D, Thompson WR, Angelopoulos TJ, Krustrup P, Mohr M, Draganidis D, Poulios A, Fatouros IG (2022). Comparative efficacy of 5 exercise types on cardiometabolic health in overweight and obese adults: A systematic review and network meta-analysis of 81 randomized controlled trials. Circ. Cardiovasc. Qual. Outcomes.

[49] Roth K, Kriemler S, Lehmacher W, Ruf KC, Graf C, Hebestreit H (2015). Effects of a physical activity intervention in preschool children. Med. Sci. Sports Exerc..

[50] Dominic D, Kishore S (2021). Effect of modified high intensity interval training on fat loss. Central Eur. J. Sport Sci. Med..

[51] Khodadadi F, Bagheri R, Negaresh R, Moradi S, Nordvall M, Camera DM, Wong A, Suzuki K (2023). The effect of high-intensity interval training type on body fat percentage, fat and fat-free mass: A systematic review and meta-analysis of randomized clinical trials. J. Clin. Med..

[52] Meng C, Yucheng T, Shu L, Yu Z (2022). Effects of school-based high-intensity interval training on body composition, cardiorespiratory fitness and cardiometabolic markers in adolescent boys with obesity: A randomized controlled trial. BMC Pediatrics.

[53] Muntaner-Mas A, Palou P (2017). Effects of high intensity interval training (HIIT) intervention amongst school adolescents. J. Phys. Educ. Health-Soc. Perspect..

[54] Kunz P, Engel FA, Holmberg HC, Sperlich B (2019). A meta-comparison of the effects of high-intensity interval training to those of small-sided games and other training protocols on parameters related to the physiology and performance of youth soccer players. Sports Med.-Open.

[55] He Z, Tian Y, Valenzuela PL, Huang C, Zhao J, Hong P, Lucia A (2018). Myokine response to high-intensity interval vs. resistance exercise: An individual approach. Front. Physiol..

[56] Marinho DA, Neiva HP, Marques L, Lopes VP, Morais JE (2022). The influence of a specific high intensity circuit training during physical education classes in children’s physical activity and body composition markers. Monten. J. Sports Sci. Med..

[57] Jurić P, Dudley DA, Petocz P (2023). Does incorporating high intensity interval training in physical education classes improve fitness outcomes of students? A cluster randomized controlled trial. Prev. Med. Rep..

[58] Hackney AC, Battaglini C (2007). The overtraining syndrome: Neuroendocrine imbalances in athletes. Br. J. Biomot..

[59] Beltran-Carrillo VJ, Devis-Devis J, Peiro-Velert C, Brown DH (2012). When physical activity participation promotes inactivity: Negative experiences of Spanish adolescents in physical education and sport. Youth Soc..

[60] Singh R, Pattisapu A, Emery MS (2020). US Physical Activity Guidelines: Current state, impact and future directions. Trends Cardiovasc. Med..

[61] Lan C, Liu Y, Wang Y (2022). Effects of different exercise programs on cardiorespiratory fitness and body composition in college students. J. Exerc. Sci. Fit..

[62] Guo L, Chen J, Yuan W (2023). The effect of HIIT on body composition, cardiovascular fitness, psychological well-being, and executive function of overweight/obese female young adults. Front. Psychol..

[63] Hargreaves M, Spriet LL (2020). Skeletal muscle energy metabolism during exercise. Nat. Metab..

[64] Meyler S, Bottoms L, Muniz-Pumares D (2021). Biological and methodological factors affecting response variability to endurance training and the influence of exercise intensity prescription. Exp. Physiol..

[65] Reuter M, Rosenberger F, Barz A, Venhorst A, Blanz L, Hecksteden A, Meyer T (2023). Does higher intensity increase the rate of responders to endurance training when total energy expenditure remains constant? A randomized controlled trial. Sports Med.-Open.

[66] Gropper H, John JM, Sudeck G, Thiel A (2023). “I just had the feeling that the interval training is more beneficial”: Young adults’ subjective experiences of physical fitness and the role of training modes. Front. Sports Act. Living.

[67] Baker JS, Quach B, Jiao J, Liang W, Gao Y (2020). Body composition matters when designing and prescribing HIIT protocols to individuals for health promotion. Phys. Act. Health.

[68] Andrade-Mayorga O, Martínez-Maturana N, Salazar LA, Díaz E (2021). Physiological effects and inter-individual variability to 12 weeks of high intensity-interval training and dietary energy restriction in overweight/obese adult women. Front. Physiol..

[69] Kruschke JK (2018). Rejecting or accepting parameter values in Bayesian estimation. Adv. Methods Pract. Psychol. Sci..

